# New technique of guidewire exchange for transanal decompression tube placement

**DOI:** 10.1111/den.14716

**Published:** 2023-11-28

**Authors:** Keita Matsumoto, Masanari Sekine, Hirosato Mashima

**Affiliations:** ^1^ Department of Gastroenterology Jichi Medical University Saitama Medical Center Saitama Japan

## Abstract

Watch a video of this article.

## BRIEF EXPLANATION

**Figure 1:**
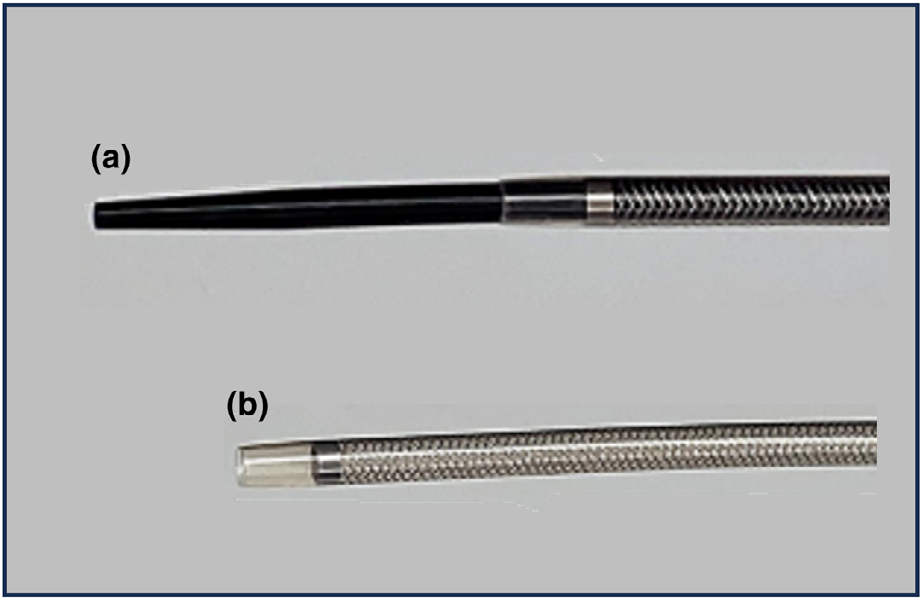
(a) Overview of the novel tapered‐tip sheath system. (b) The outer sheath after the removal of the inner catheter.

**Figure 2:**
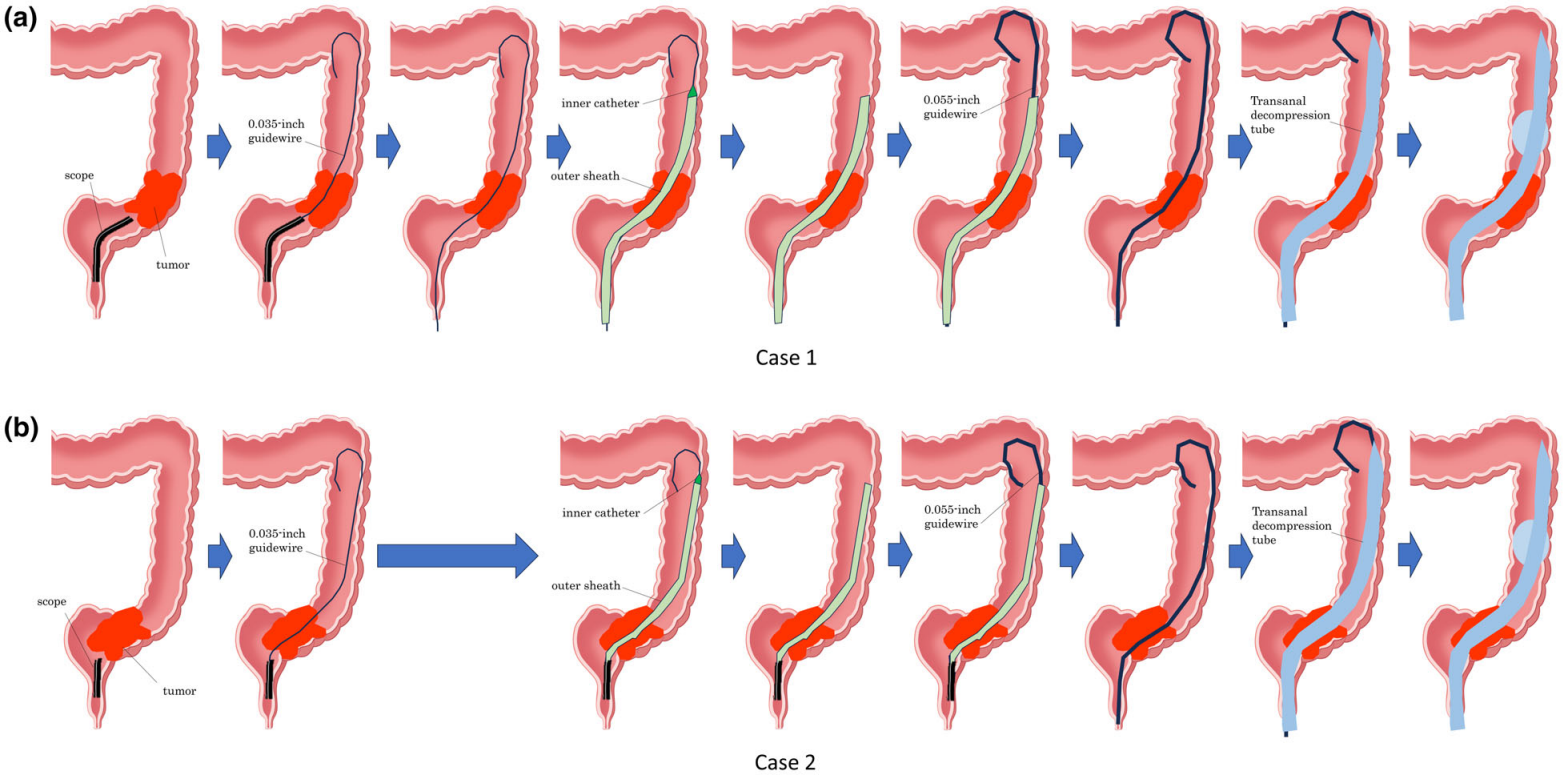
A schematic illustration of the method. (a) Case 1: The endoscope could not pass through the stenosis caused by sigmoid colon cancer. A 0.035‐inch guidewire could pass through the stenosis. The 0.035‐inch guidewire was kept in place and the endoscope was removed. A novel tapered‐tip sheath system could pass through the stenosis. The inner catheter was removed. A 0.055‐inch guidewire was placed through the outer sheath. The 0.055‐inch guidewire was kept in place and the outer sheath was removed. A transanal decompression tube (TDT) was placed over the 0.055‐inch guidewire. The 0.055‐inch guidewire was removed and the balloon of the TDT was inflated. (b) Case 2: The endoscope could not pass through the stenosis caused by rectal cancer. A 0.035‐inch guidewire could pass through the stenosis. A novel tapered‐tip sheath system inserted through the scope could pass through the stenosis without removing the scope. The inner catheter was removed. A 0.055‐inch guidewire was placed through the outer sheath. The 0.055‐inch guidewire was kept in place and the endoscope and the outer sheath was removed. A TDT was placed over the 0.055‐inch guidewire. The 0.055‐inch guidewire was removed and the balloon of the TDT was inflated.

Drainage is an important and frequently used treatment for gastrointestinal diseases. When a drainage tube is placed, it is often preceded by the insertion of a guidewire. Especially in transanal decompression tube (TDT) placement for malignant colorectal stenosis, a thick guidewire with appropriate rigidity is necessary. However, thick guidewires tend to have a poor seeking ability, and passing through a long and strong stenosis is often a challenge. Recently, Matsumori *et al*.^1^ reported that a novel tapered‐tip sheath system (EndoSheather; Piolax Medical Devices, Kanagawa, Japan; Fig. 1) was useful for performing transpapillary biopsies for the diagnosis of biliary stricture. In this report we describe the use of this method for the placement of a TDT (Dennis Colorectal Tube; Covidien, Tokyo, Japan). After endoscopically implanting a 0.035‐inch guidewire for biliopancreatography, which has a high seeking ability, we could easily replace it with a thicker guidewire, resulting in the successful placement of a TDT (Fig. 2). The endoscope (PCF‐H290ZI, GIF‐Q260J; Olympus Medical Systems, Tokyo, Japan) was advanced to the front of the stenosis. An endoscopic retrograde cholangiopancreatography catheter (Shoren; Kaneka Medix, Tokyo, Japan, Swish; Boston Scientific, Marlborough, MA, USA) and a 0.035‐inch guidewire (SeekMaster; Piolax Medical Devices) were used to pass through the stenosis. The use of an angle‐type 0.035‐inch guidewire makes it relatively easy to pass through the stenosis. In Case 1, the guidewire was kept in place and the endoscope was removed. The novel tapered‐tip sheath system was passed through the stenosis, over the guidewire. The inner catheter was removed and a thicker guidewire was placed inside the outer sheath. In Case 2, we could exchange the guidewire with this system directly through the scope before removing the scope. With either method, a 0.055‐inch guidewire can be easily exchanged using the novel sheath. Finally, a TDT was easily placed over the inserted thick guidewire.

Authors declare no conflict of interest for this article.

## INFORMED CONSENT

The subject of the case report provided informed consent to publish the included information.

## Supporting information


**Video S1** Transanal decompression tube placement for malignant colorectal stenosis via guidewire exchange using a novel tapered‐tip sheath system.
